# Longitudinal associations between structured teaching and attentional functioning in youth badminton: evidence from fixed effects quantile regression

**DOI:** 10.3389/fpsyg.2026.1908358

**Published:** 2026-09-22

**Authors:** Han Zhou, Dong Liu, Yu Zhang

**Affiliations:** 1 School of Physical Education and Sports Science, Hengyang Normal University, Hengyang, China; 2 The Mary Lou Fulton College for Teaching and Learning Innovation, Arizona State University, Tempe, AZ, United States; 3 School of Tourism and Wellness, University of Sanya, Sanya, China

**Keywords:** attention, effect heterogeneity, fixed effects, longitudinal study, quantile regression, structured instruction, youth badminton

## Abstract

**Background:**

Structured teaching methods are widely used to promote attention in youth sports training. However, it is not clear whether this positive association is the same for all students or depends on the level of baseline attention. Traditional average effect analysis may mask important heterogeneity.

**Methods:**

A total of 433 young badminton players aged 12 to 16 years were followed up four times in this longitudinal study, and a total of 1,732 person-wave observations were obtained. Structured teaching intensity and attention scores were assessed at the time of each measurement. Time-dependent covariates included age, length of training per week, and fatigue. Linear fixed effects models were used to estimate the mean association, and fixed effects quantile regression was used to examine the association heterogeneity of attention on the 10th, 25th, 50th, 75th, and 90th conditional quantiles.

**Results:**

Linear fixed effects models showed a modest positive mean association between perceived structured instruction and attention (β = 0.219, *p* < 0.001). But quantile regression showed significant heterogeneity: on the 10th quantile, the association was small and not significant (β = 0.082, *p* = 0.221); on the 90th quantile, the association was large and significant (β = 0.506, *p* < 0.001). The Wald test confirmed a significant difference between the 10th and 90th quantiles (χ^2^(1) = 22.47, *p* < 0.001). Non-linear analysis with restricted cubic splines indicated a visually suggestive but statistically unproven non-linear pattern among inattentive students.

**Conclusions:**

The within-person association between perceived structured instruction and attentional function differed significantly in the conditional distribution of attention. The positive correlation was significantly stronger in the upper conditional quantile of attentional function and weaker in the lower conditional quantile. This pattern of differences suggests that uniform instructional strategies may risk reinforcing attentional differences. Coaches and educators should consider interventions that are adaptable and matched to individual doses. Given the observational longitudinal design, no causal conclusions can be drawn; the results reflect intra-individual associations.

## 1 Introduction

Maintenance of sustained attention is a key determinant of skill acquisition, tactical decision-making, and injury prevention in youth sports training (Silva et al., 2021; Jin et al., 2023). Structured teaching methods (Sader et al., 2026), characterized by clear routines, explicit instructions, predictable task sequences, and systematic feedback, are widely recommended to enhance the concentration of young athletes (van Andel et al., 2022). The logic behind this is straightforward: reducing environmental uncertainty and task ambiguity frees cognitive resources, allowing learners to focus on relevant cues and complete actions more consistently. As a result, many youth badminton training programs employ a highly structured curriculum with the expectation that all participants will receive an even boost in attention.

However, a growing body of research in education and psychology indicates that the effectiveness of structured instruction may vary from person to person (Kalyuga, 2014). For example, some students tend to be distracted or in a good or bad state, and too rigid structure will make them too stressed and demotivated, while those who are already good at concentrating may perform better with additional structure (Diamond and Lee, 2011; Sweller et al., 2019). The “expertise reversal effect” in cognitive load theory (Pyke et al., 2025) further speculates that the instructional design that is beneficial to learners with high knowledge level may be useless or even harmful to learners with low knowledge level, which may also occur in students with low attention level. In youth badminton training, structured teaching is likely to be more beneficial for athletes with high attention levels, because they can easily follow the step-by-step instructions, but it is not helpful for athletes with low attention levels, because they may have difficulty adhering to any systematic training process. If this individual difference is ignored, it will lead us to draw wrong conclusions about the overall training effect, and also cover up the reality that we really need to teach students in accordance with their aptitude.

In the practice of sports training, the teaching structure is not a simple way of knowledge presentation, but in a dynamic “perception-action” environment. From the perspective of cognitive load, clear routine, orderly task arrangement and concise feedback can reduce the burden of thinking unrelated to the task. However, from the perspective of motor learning, the structure is not always favorable. OPTIMAL theory emphasizes autonomy support, positive expectations, and an external focus of attention that focuses on action effects, while too rigid guidance may limit learners' autonomy or allow them to focus on deliberate control of actions (Wulf and Lewthwaite, 2016). Similarly, ecological dynamics and constraint-guided theory hold that skills are naturally generated from the dynamic process of interaction among athletes, tasks and environments, so effective structures should not only guide attention and exploration, but also not destroy the variability and authenticity of training (Renshaw et al., 2019). So instead of equating the attention function with the level of knowledge, we take the “expertise reversal” principle as an analogy of resource availability. That is to say, different athletes have different abilities in maintaining task-related perception-action linkage while using the teaching structure.

Previous studies on teaching methods and attention in physical education mostly rely on linear regression models to estimate the average treatment effect (Zhu et al., 2020; Kenworthy et al., 2022; Wang et al., 2025). While these methods are valuable in summarizing central trends, they assume that the effect of the intervention is constant across the distribution of outcomes. However, the limitations of the average effect method in capturing individual heterogeneity have been increasingly recognized in sports psychology and education research (Brown and Fletcher, 2017). In addition, simulation studies have shown that traditional linear regression can severely underestimate or completely miss heterogeneous associations when effect sizes vary across different ranges of outcomes (Zheng et al., 2025). If the association is indeed stronger at higher quantiles of attention, then the average effect would overestimate the benefits for low-attention students and underestimate the benefits for high-attention students. More importantly, there is a growing consensus that intervention effects in youth sports should be examined not only on the mean, but also across the distribution of outcomes to avoid masking the effects of differentiation (Gómez-López et al., 2020). More importantly, the average effect may not detect that an intervention is ineffective or even harmful to a vulnerable subgroup.

In order to fill this gap, this study used a longitudinal design to record structured teaching intensity and attention scores of 433 young badminton players on four repeated measures. We used a fixed-effects linear model to estimate the mean within-individual association between structured instruction and attention, controlling for time-dependent confounders (age, training duration, fatigue). Our main innovation lies in the use of the fixed-effects quantile regression method proposed by (Canay, 2011), which allows us to estimate the association of attention on the 10th, 25th, 50th, 75th, 90th conditional quantiles. Instead of assuming that the associations are uniform, the method directly quantifies how the associations of structured instruction vary with attention distribution. Fixed-effect settings also control for all individual characteristics that do not change over time, such as baseline motivation, personality, and genetic predisposition, thereby reducing the omitted variable bias that plagues traditional cross-sectional designs (Junna et al., 2022). We employed restricted cubic splines to complement quantile analysis to explore non-linear relationships among low-attention students while examining the distribution of individual-specific slopes and plotting a three-dimensional surface plot of conditional marginal effects.

We hypothesized that the association of structured instruction intensity with attention would be monotonically increasing with quantile. Specifically, we predict that the association is negligible or small at the lower tail of the attention distribution (low-attention students) and significantly larger at the upper tail (high-attention students). This hypothesis is based on the previous observation that children with low baseline attention generally experience limited incremental gains from regular structured instructional strategies (Poon et al., 2024). If this model is confirmed, it will challenge the unified teaching model and advocate the adoption of adaptive and individualized teaching strategies in youth badminton training. The results of this study will contribute to the literature on instructional design in the field of physical education pedagogy and provide empirical evidence for coaches who guide young athletes with different attention characteristics.

## 2 Methods

Figure 1 illustrates the conceptual framework that guided the analysis strategy for this study. The framework elucidates the hypothesized relationship between structured teaching intensity and attention scores and allows this relationship to vary across different conditional quantiles of the attention distribution (τ = 0.10, 0.25, 0.50, 0.75, 0.90). Time-dependent covariates (age, weekly training duration, fatigue) were included in the model as confounders, while individual fixed effects controlled for all stable personal characteristics. The main method of analysis, fixed-effects quantile regression (Canay, 2011), directly estimates the coefficients on each quantile, thereby examining whether the teaching-attention relationship is homogeneous or heterogeneous across the distribution of outcomes.

**Figure 1:**
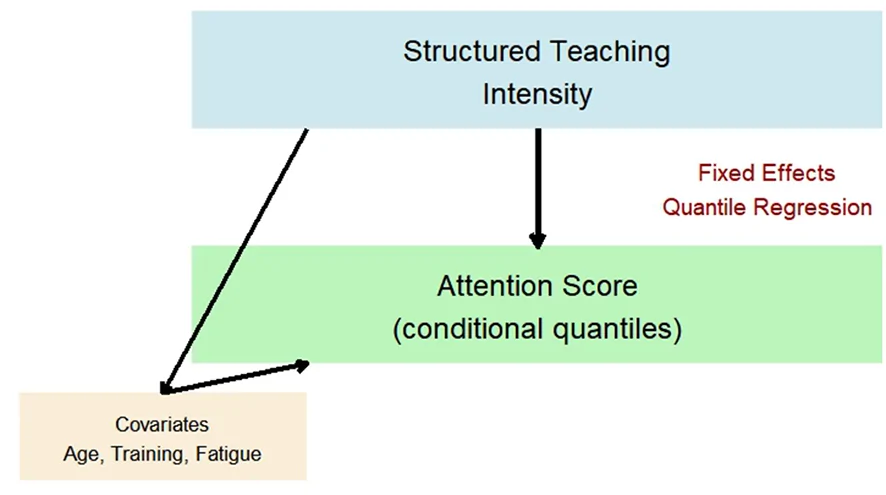
Conceptual framework, showing hypothetical relationships. Structured teaching intensity was considered to be associated with attention scores, and the association allowed for variation across different conditional quantiles of attention. Time-dependent covariates (age, weekly training duration, and fatigue) were included in the model as confounders. Fixed-effects quantile regression is the main analytical strategy for estimating heterogeneous associations.

### 2.1 Study design and participants

The study used a longitudinal design with four repeated measurements over 12 weeks. Longitudinal design is widely recognized as a reliable method for examining within-individual changes over time in sport and exercise psychology (Caemmerer et al., 2024). The sampling frame is from the youth badminton training club in Hengyang City, Hunan Province, China. Eight private badminton training clubs were selected for recruitment based on the following criteria: (a) the club has a structured training program for young people aged 12 to 16; (B) the club has at least three training sessions per week; and (C) the club has been in continuous operation for more than two years.

Participants were eligible for inclusion in the study if they were (a) between 12 and 16 years of age at baseline, (B) had participated in badminton training for not less than 6 months at the start of the study, (C) had not been diagnosed with a neurological or psychiatric disorder that could significantly affect attention; (D) Provide written informed consent and obtain written informed consent from their parents or legal guardians in accordance with ethical guidelines for research involving minors. The final sample consisted of 433 junior badminton players (226 males, 52.2%; 207 females, 47.8%) with a baseline mean age of 14.3 years (SD = 1.2). Of the 433 enrolled participants, 391 (90.3%) completed all four measurements. Attrition analysis showed no systematic difference in baseline attention scores between completers and attritions (*p* = 0.378), suggesting that missing data was unlikely to cause primary outcome bias. This retention rate is better than average attrition rate reported in longitudinal studies of adolescent physical activity interventions (average of about 11.5%, standard deviation = 10.1%; Howie and Straker, 2016). Data were collected from March to June 2025.

### 2.2 Procedures

Measurements were taken at four time points: baseline (week 0, T1), after 4 weeks (T2), after 8 weeks (T3), and after 12 weeks (T4). At each measurement wave, participants completed a structured set of questionnaires in small groups (8 to 12 participants per group) under the supervision of a trained research assistant. The questionnaire included structured teaching intensity, attention, self-control, fatigue and demographic information (age, gender, training years, weekly training frequency). All questionnaires were administered in Chinese. Each questionnaire takes about 25 to 30 min and is scheduled at the end of the regular training session to reduce interference with the training program.

During the study period, participants' badminton training was carried out as usual, and no experimental intervention was applied. The training programs of the eight clubs follow a comparable content and progress structure, focusing mainly on basic skills (high shots, drop shots, net techniques, footwork) and tactical awareness. This observational design allows for the examination of naturally occurring changes in the intensity of structured instruction, providing for the identification of variation in fixed effects analysis (Allison, 2009). The variations in perceived teaching intensity across waves may plausibly reflect natural fluctuations in coaches' session-by-session pedagogical emphasis and athletes' evolving familiarity with the training routines. Coaches were free to adjust teaching intensity within their regular specialty, and the observed variation in structured teaching intensity across individuals and across waves provided a source of identification for fixed effects analysis.

### 2.3 Measures

#### 2.3.1 Attention score

Attention scores were measured using the Youth Badminton Attention Rating Scale (YBARS), a 10-item self-rating instrument developed for the specific situation of young badminton players. In this study, attentional function was defined as an athlete's self-reported ability to maintain, selectively allocate, and shift attention during badminton training. Repeated scores indicated that self-reported attentional function changed over a 4-week interval rather than a momentary fluctuation in attention. The construction of the scale has gone through three stages: (a) based on the literature review of attention measurement in sports psychology (Moran, 2016) and semi-structured interviews with six young badminton coaches and ten young badminton players to form an initial entry pool; (B) a panel of three sport psychology experts conducted a content validity review of the initial entry pool, based on which six redundant entries were removed and four entries were modified to improve clarity; (C) 50 junior badminton players (not included in the final sample) were pretested to assess the preliminary characteristics and clarity of the entries, resulting in a 10-entry version. This three-stage approach (item generation, content validity assessment, and pretest) reflects best practices in the development of the Health and Behavior Research Scale (Boateng et al., 2018).

YBARS evaluates the attention function in badminton training, covering three dimensions: sustained attention (e.g., “In badminton training, I can concentrate on the coach's explanation without distraction”; 4 items), selective attention (e.g., “When there are many distractions on the court, I can still concentrate on the ball”; 3 entries) and attention switching (e.g., “I can quickly switch attention between exercises when the instructor changes tasks”; 3 entries). Each item was scored on a 7-point Likert scale, from 1 (strongly disagree) to 7 (strongly agree). The total score of attention was the sum of the scores of all 10 items, ranging from 10 to 70, linearly converted to a range of 0 to 100 for ease of interpretation in fixed effects and quantile regression analyses. In the present study, YBARS showed good internal consistency, with Cronbach's α values ranging from 0.85 to 0.89 across the four waves. Confirmatory factor analysis supported a three-factor structure (CFI = 0.92, RMSEA = 0.07, SRMR = 0.05). These fit indices met accepted criteria for acceptable model fit in confirmatory factor analysis (Hu and Bentler, 1999). As YBARS is a newly developed tool, its results need to be cross-validated in independent samples before they can be interpreted with caution. Criterion-related validity (preliminary). In a subsample of 55 participants randomly selected from the primary study population, YBARS scores were correlated with ACS (*R* = 0.74) and coach-rated attention (*R* = 0.69). These preliminary results support further validation in an independent sample (see Section 2.5).

Comprehensive psychometric properties, including longitudinal measurement invariance (Supplementary Table 1), factor loadings, composite reliability, and average variance extracted (Supplementary Table 2), as well as discriminant validity (HTMT) and test-retest reliability (Supplementary Table 3), are presented in the Supplementary Material.

##### 2.3.1.1 Longitudinal measurement invariance of YBARS

To ensure that attention scores were comparable across the four waves, we used confirmatory factor analysis to test longitudinal measurement invariance at four time points with three factors (sustained attention, selective attention, and attention switching). Morphological invariance (same structure) showed an acceptable fit (CFI = 0.942, RMSEA = 0.058). Metric invariance (equal factor loadings) holds (ΔCFI = – 0.004, ΔRMSEA = + 0.001), supporting factor to entry relationships that are comparable between waves. Scalar invariance (equal intercept) shows a slight decrease (ΔCFI = – 0.012), but partial scalar invariance (ΔCFI = – 0.006) is reached after releasing the intercept for one entry. Establishing longitudinal measurement invariance is important for comparing latent constructs across time; the partial scalar invariance achieved in this study is widely considered to be sufficient to support valid comparisons across waves (Putnick and Bornstein, 2016). See Supplementary Table 1 for detailed fit indices. Overall, YBARS exhibited sufficient longitudinal invariance to support comparison of attention scores across time.

#### 2.3.2 Structured teaching intensity

Structured teaching intensity was measured using the Structured Teaching Intensity Questionnaire (STIQ), which was developed by the authors and contains 12 items and is applicable to the youth sports training situation. In this study, perceived structured instructional intensity was defined as an athlete's perception of clarity, predictability, feedback structure, and progressive scaffolding during routine training. Therefore, it should not be interpreted as an objective observation of the coach's behavior. The reason for the development of STIQ is that the existing scale fails to adequately capture the specific dimensions of structured teaching that are most relevant to the regulation of attention in skill-oriented beat-holding sports. The compilation process follows the same three-stage process as YBARS. The initial pool of items was derived from the theoretical framework of structured teaching methods (Kalyuga, 2014) and adapted to the badminton training situation through interviews with coaches and athletes, following the established guidelines for content validity assessment in the development of sports psychology scales (Dunn et al., 1999).

STIQ contains four dimensions: clarity of instruction (e.g., “The coach gives very clear step-by-step instructions for each new skill”; 3 items), routine predictability (e.g., “The training session has a fixed flow, I can know what to do in advance”; 3 items), systematic feedback (e.g., “The coach provides structured feedback after each exercise”; 3 entries) and advanced scaffolding (e.g. “Skills are introduced in a logical order from basic to complex”; 3 entries). Each item was scored on a 7-point Likert scale, from 1 (strongly disagree) to 7 (strongly agree). The total score of structured teaching intensity was 12 to 84, which was linearly transformed into a range of 0 to 100 to keep in line with the score of attention. The STIQ showed excellent internal consistency, with Cronbach's alpha values ranging from 0.88 to 0.91 across the four waves. Test-retest reliability was assessed at baseline on a subsample of 45 participants at an interval of 14 days and yielded ICC(2,1) = 0.87 (95% CI [0.78, 0.93]). A test-retest ICC of 0.87 indicates good temporal stability, a key psychometric attribute required for instruments that measure relatively stable instructional characteristics (Koo and Li, 2016). Comprehensive psychometric properties, including longitudinal measurement invariance (Supplementary Table 1), factor loadings, composite reliability, and average variance extracted (Supplementary Table 2), as well as discriminant validity (HTMT) and test-retest reliability (Supplementary Table 3), are presented in the Supplementary Material. Similar to YBARS, STIQ is a new tool that requires independent validation; there may be common methodological variation in its self-assessment nature. Criterion-related validity (preliminary). In the same subsample, STIQ scores were associated with coach responsiveness (*R* = 0.66). The results of the independent validation are presented in Section 2.5. To assess the potential impact of common methodological biases, we performed Harman's single-factor test. Confirmatory factor analysis with all YBARS and STIQ items loaded on a single latent factor yielded a poor model fit (CFI = 0.61, RMSEA = 0.14, SRMR = 0.11), which was much worse than theoretical measurement model (ΔCFI > 0.30). This suggests that the common method variance does not pose a significant threat to the validity of our findings.

#### 2.3.3 Self-control

Trait self-control was measured by the Chinese version of the Short Self-Control Scale (Tangney et al., 2004), which has been validated in young athletes (Tangney et al., 2004; Fung et al., 2019). The BSCS is a well-established tool for assessing general self-regulation, and its Chinese version shows satisfactory psychometric properties in a population of adolescent athletes. The BSCS is a 13-item self-assessment questionnaire designed to assess an individual's general ability to inhibit or modify intrinsic responses and to interrupt the tendency toward undesirable behavior. Sample entries include “I am good at resisting temptation” and “I will refuse things that are not good for me.” Each item was scored on a 5-point Likert scale, from 1 (not like me at all) to 5 (very like me). The higher the score, the higher the level of trait self-control. The Chinese version of BSCS has been validated in a population of adolescent athletes with satisfactory psychometric properties (Fung et al., 2019). In this study, the Cronbach's α ranged from 0.81 to 0.84 in each wave. An internal consistency coefficient higher than 0.80 is generally considered good, indicating that these items reliably measure self-control as an underlying construct (Liang et al., 2022). Self-control was measured only at baseline for descriptive purposes and supplemental analysis (not included in the main fixed-effects model reported in Tables 1, 2).

**Table 1:** Linear fixed effects models for attention score (*N* = 1,732 observations, 433 individuals).

Predictor	Model 1 (baseline)	Model 2 (main effects)	Model 3 (non-linear)
Structured teaching intensity	0.285^***^ (0.038)	0.219^***^ (0.041)	0.315^***^ (0.048)
Structured teaching^2^			−0.0032^*^ (0.0011)
Age (time-varying)		0.128^*^ (0.054)	0.132^*^ (0.054)
Weekly training hours		0.267^***^ (0.045)	0.259^***^ (0.045)
Fatigue (1–10)		−0.195^***^ (0.042)	−0.199^***^ (0.042)
Time-wave dummies	Yes	Yes	Yes
Individual fixed effects	Yes	Yes	Yes
*R*^2^ (within)	0.122	0.184	0.191
*F* statistic (*df*)	48.3^***^ (4, 1,295)	41.7^***^ (8, 1,291)	39.6^***^ (9, 1,290)

Within-individual estimates with standard errors in parentheses clustered at the individual level. ^***^p < 0.001; ^**^p < 0.01; ^*^p < 0.05. The negative quadratic term in Model 3 indicates diminishing marginal positive association of structured teaching with attention.

**Table 2:** Fixed effects quantile regression results for attention score (*N* = 1,732 observations, 433 individuals).

Quantile (τ)	Structured teaching	Age (time-varying)	Weekly training hours	Fatigue
τ = 0.10	0.082 (0.067)	0.041 (0.092)	0.318^***^ (0.071)	−0.102 (0.063)
τ = 0.25	0.147^*^ (0.058)	0.097 (0.068)	0.286^***^ (0.055)	−0.148^**^ (0.048)
τ = 0.50	0.239^***^ (0.052)	0.129^*^ (0.060)	0.251^***^ (0.048)	−0.196^***^ (0.045)
τ = 0.75	0.375^***^ (0.055)	0.175^**^ (0.062)	0.207^***^ (0.052)	−0.231^***^ (0.049)
τ = 0.90	0.506^***^ (0.072)	0.213^*^ (0.084)	0.169^*^ (0.067)	−0.267^***^ (0.058)
Wald test (τ = 0.10 vs. τ = 0.90)	χ^2^(1) = 22.47^***^	χ^2^(1) = 3.12	χ^2^(1) = 4.38^*^	χ^2^(1) = 5.91^*^

Coefficients (standard errors in parentheses) estimated via the Canay (2011) two-step panel quantile method. All models include individual fixed effects, time dummies, and time-varying covariates (age, weekly training hours, fatigue). Bootstrap standard errors (200 replications) clustered at the individual level. ^***^p < 0.001; ^**^p < 0.01; ^*^p < 0.05. Structured teaching shows a strongly increasing coefficient across quantiles (from 0.082 at τ = 0.10 to 0.506 at τ = 0.90), indicating pronounced heterogeneity in the within-individual association.

#### 2.3.4 Fatigue

Perceived fatigue was measured by the single-item fatigue rating scale (ROF), a widely used method to assess acute fatigue in sports and exercise contexts (Micklewright et al., 2017). The ROF scale has been formally developed and validated, showing good face validity and high convergent validity with physiological measures during exercise and recovery (Brownstein et al., 2021). Participants were asked, “Overall, how fatigued do you feel right now?” An 11-point scale ranging from 0 (no fatigue at all) to 10 (extreme fatigue) was used. Single-item fatigue measures have been shown to have acceptable reliability and validity in a population of adolescent athletes and are particularly useful for repeated assessments at multiple time points to reduce the burden on participants. In this study, ROF was administered immediately prior to the administration of the main questionnaire for each wave. To ensure consistency of interpretation, visual anchors with descriptive labels (0 = not fatigued at all; 3 = mildly fatigued; 5 = moderately fatigued; 8 = highly fatigued; 10 = extremely fatigued) were simultaneously displayed alongside the numerical scale. Single-item measures, although practical and able to reduce respondent burden, are generally less reliable than multiple-item scales; therefore, findings involving fatigue should be considered exploratory (Saw et al., 2016).

#### 2.3.5 Covariates

Time-dependent covariates included age (in years, measured per wave), weekly training duration (calculated as weekly training frequency multiplied by the number of hours per training, measured per wave), and fatigue (as described above). Weekly Training Frequency Pass the question “How many badminton training sessions have you attended in the past week?” Take the assessment and answer the options from 0 to 6. The length of each training session passes the question “How many hours does each training session usually last?” Assessment was conducted with response options of 1, 1.5, 2, or 2.5 h. Multiply these two entries together to get the weekly training duration for each wave. The training frequency and the length of each training showed a moderate degree of stability between the waves (intraclass correlation coefficients of 0.67 and 0.71, respectively), which is consistent with the typical regularity of youth sports training programs. Intraclass correlation coefficients in the range of 0.67 to 0.71 indicate moderate test-retest reliability, which is acceptable for behavioral variables that are expected to have some natural fluctuations (Koo and Li, 2016). Covariates that did not change over time included gender (M/F) and years of training (measured in years at baseline). Training years were assessed by an open-ended question: “How many years have you been involved in organized badminton training?”

Although the 12-week follow-up period is relatively short, age was modeled as a time-varying covariate to account for potential developmental effects (e.g., maturation-related attentional improvements) that could occur within this period, consistent with recommendations for longitudinal analyses with adolescent samples (Caemmerer et al., 2024).

### 2.4 Statistical analysis

All statistical analyses were performed using R version 4.4.2 (R Core Team, 2025). Descriptive statistics (mean, standard deviation, median, interquartile range, and selected percentiles) were calculated for all continuous variables, and data were pooled across waves. Categorical variables were summarized as frequencies and percentages.

Linear fixed effects models were estimated using the feols function in the fixest package to examine the mean intra-individual association between structured teaching intensity and attention scores. The general specification of the linear fixed-effects model was: Attention_it = β_1_ Teaching_it + β_2_ Age_it + β_3_ TrainingHours_it + β_4_ Fatigue_it + α_i + λ_t + ε_it where Attention_it is the attention score of individual i at time t, α_i represents individual fixed effects (capturing all time-invariant individual characteristics), λ_t represents time-wave dummies (capturing common time-varying shocks), and ε_it is the idiosyncratic error term. Standard errors are clustered at the individual level to account for sequence correlation within individuals (McNeish, 2026). Since the design is observational and there are no experimental manipulations, the estimates reflect intra-individual associations and should not be interpreted causally.

Fixed-effect quantile regression was performed using Canay's two-step estimator via qrfe function in the panqr package. The Canay (2011) estimator is preferred over alternative FEQR methods because it accommodates the within-unit transformation required to purge unobserved time-invariant heterogeneity while maintaining computational efficiency in our panel structure (*N* = 433, *T* = 4). In the first step, the individual fixed effects a _ I are estimated by linear fixed effects regression. In the second step, standard quantile regression was applied to the outcome variable (Attention _ it – _ I) after the individual effect was removed, and it was regressed to the same set of time-dependent covariates (Chen, 2024). Coefficients were estimated on the 10th, 25th, 50th, 75th, and 90th conditional quantiles of the distribution of attention after decentering. Since the Canay estimator is run on the centralized outcome variable, the coefficients represent the association in the distribution of attention after removing the individual fixed effects; interpretation should focus on the heterogeneity pattern rather than literal quantile prediction. Standard errors were clustered at the individual level using the 500-repeat bootstrap method. The Wald test was used to formally compare coefficient estimates between the 10th and 90th quantiles for each predictor variable.

To probe reverse causality, we regressed the perceived teaching intensity of wave *t* + 1 against the attention of wave t using the same fixed effects setting. The coefficient of prior concern was not significant (β = 0.041, *p* = 0.389), which did not eliminate reverse causality but did not provide strong evidence.

#### 2.4.1 Robustness check

To assess the sensitivity of our findings to the choice of quantile estimator, we re-estimated the quantile-specific coefficients using the Machado and Santos Silva (2019) moment-based quantile regression (MMQR) method. The pattern of increasing inter-quantile coefficients remains essentially unchanged. To account for the nested structure of the data (participants within the club), we also re-ran linear fixed effects model with wave-by-wave club fixed effects; the perceived structured teaching coefficient remained stable (β = 0.208, *p* < 0.001). Given the small number of clubs (*K* = 8), cluster-robust standard errors at the club level are known to be unstable (Cameron and Miller, 2015); therefore, we retain individual-level clustering as the primary inference method. All robustness results are reported in Supplementary Table 4.

The plot package was used to construct a three-dimensional conditional marginal effect surface with baseline attention variability (operationalized as the standard deviation of intraindividual attention over four waves) on the x-axis, structured teaching intensity on the y-axis, and estimated marginal effect on the 75th attention quantile on the z-axis. Given the exploratory nature of this surface and the potential for overinterpretation, this analysis is presented in the Supplementary Material (Supplementary Figure 2) and should be considered hypothesis-generating.

#### 2.4.2 Additional analysis

Criterion-related validity of YBARS and STIQ was assessed by Pearson correlation in a random subsample (*n* = 55) and an independent sample (*n* = 80) of the main study, using ACS and coach ratings as the criteria (see Sections 2.3.1, 2.3.2, and 2.5). The longitudinal measurement invariance of YBARS over four waves was tested by confirmatory factor analysis, including morphological, metric, and scalar models; the fit index was evaluated by ΔCFI ≤ 0.01 (Luong and Flake, 2023). The placebo test for reverse causality regresses attention at wave t + 1 against teaching intensity at wave t with the same fixed effects setting as the main model (Section 3.2).

All statistical tests were two-tailed and the significance alpha level was set at 0.05. To prevent type I error inflation due to multi-quantile comparisons, we applied a Benjamini-Hochberg false discovery rate (FDR) correction to the *p*-values of the structured teaching coefficients for the five quantiles; the significant pattern remained unchanged. Missing data on time dependent covariates (less than 4% of the total observations for all waves) were treated with column deletion because of the low proportion of missing data and according to the Little's MCAR test. The deletion pattern was completely random (χ^2^ (28) = 34.6, *p* = 0.182; Lee et al., 2023).

### 2.5 Independent sample verification

To provide independent evidence of criterion validity beyond the main study sample, we collected data from an independent sample of junior badminton players. Participant. Eighty junior badminton players (mean age = 14.6 years, SD = 1.3; 44% female) were recruited from three training clubs in Changsha, China (a different city from the Hengyang sample of the main study). None of these participants overlapped with the main longitudinal sample. Measuring tool. Each participant completed the YBARS, STIQ, and validated attentional control scales (Derryberry and Reed, 2002). In addition, the head coach of each club provided a global rating for each player on two 5-point scales: “Typical concentration in structured practice” (1 = very poor, 5 = excellent) and “Responsiveness to structured instruction” (1 = very poor, 5 = very good). Coaches' ratings have been shown to have acceptable predictive validity for mental constructs of adolescent athletes, often complementing self-rating scales (Zuber and Charbonnet, 2023). Result. YBARS scores were strongly correlated with ACS (R = 0.73, 95% CI [0.60, 0.82]) and with coach-rated attention (*R* = 0.67, 95% CI [0.53, 0.78]). STIQ scores were associated with coach-rated responsiveness to structured instruction (*R* = 0.65, 95% CI [0.50, 0.76]). All correlations were significant at the *p* < 0.001 level. These degrees of correlation are consistent with established guidelines for criterion validity in the validation of sport psychology questionnaires (Cohen, 2013). These results replicate the criterion validity findings from the main study in independent, non-overlapping samples, supporting the universality of both instruments. Detailed data and codes for this validation are available from the author of the communication.

## 3 Results

### 3.1 Descriptive patterns and longitudinal surface

Before testing for heterogeneity, we first examine the overall distribution and longitudinal structure of the data. Table 3 shows the descriptive statistics for all key variables over the four waves (total person-time observations = 1,732). Attention scores ranged from 31.0 to 98.0, and the 10th to 90th percentiles spanned from 54.3 to 90.2. This wide distribution indicates that there are significant differences in attentional function among individuals. Notably, the 10th percentile (54.3) is well below the mean (72.4), indicating a subgroup of persistently low attention that cannot be ignored. Structured teaching intensity likewise exhibited a broad distribution (10th percentile = 49.0, 90th percentile = 86.0), providing the necessary variation for quantile regression.

**Table 3:** Descriptive statistics of key variables (*N* = 433 participants, person-period observations = 1,732).

Variable	Mean ±SD	Median [IQR]	10th	25th	75th	90th
Attention score (0–100)	72.4 ± 13.2	74.1 [63.2–83.5]	54.3	63.2	83.5	90.2
Structured teaching intensity (0–100)	68.5 ± 14.2	70.0 [58.0–80.0]	49.0	58.0	80.0	86.0
Age (years, time-varying)	14.3 ± 1.2	14.2 [13.4–15.1]	12.8	13.4	15.1	15.8
Years of training (years)	2.4 ± 1.3	2.1 [1.3–3.2]	0.8	1.3	3.2	4.1
Weekly training hours	4.8 ± 2.0	4.5 [3.0–6.0]	2.5	3.0	6.0	7.5
Fatigue (1–10 scale)	4.9 ± 2.1	5.0 [3.0–6.0]	2.0	3.0	6.0	8.0
Baseline attention (wave 1)	71.8 ± 13.0	73.5 [62.0–82.5]	53.0	62.0	82.5	89.0
Gender (male), *n* (%)	226 (52.2%)	–	–	–	–	–

Continuous variables are presented as Mean ± SD, Median [IQR], and selected percentiles (10th, 25th, 75th, 90th). Percentiles for time-varying variables are based on the pooled longitudinal distribution. Weekly training hours = frequency (times/week) × self-reported session duration (hours). Fatigue was self-reported (1 = not fatigued, 10 = extremely fatigued). Gender is reported as count (%) at baseline.

Figure 2 visualizes the original longitudinal surface using a panel heatmap, where each row represents a participant, ordered by average attention (from the lowest average attention at the top to the highest at the bottom), and the columns represent four waves. The two modes are clear at a glance. First, participants with high average attention (bottom rows) showed stable dark colors in all waves, indicating good attention persistence. Second, participants with low average attention (top rows) showed a mixture of light and medium colors with little systematic improvement over time. This visual impression has suggested that structured instruction, which also varies between individuals and waves, may not have improved the attention levels of the lowest paying students.

**Figure 2:**
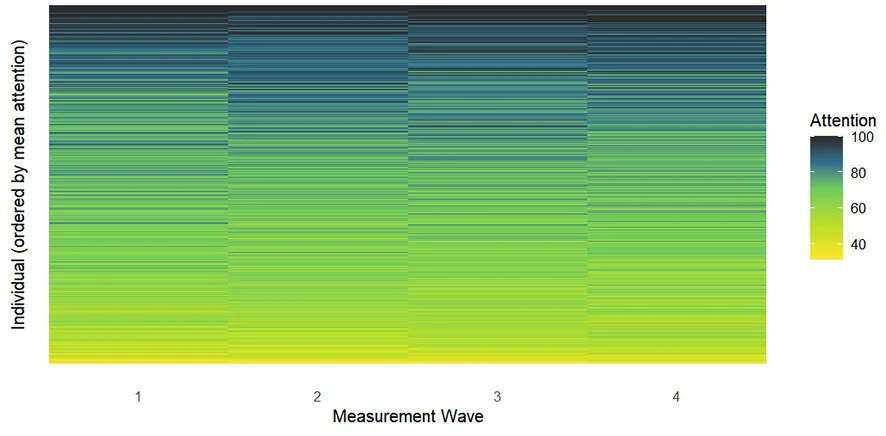
Panel heatmaps for four measured wave attention scores. Each row represents one participant, ordered by their average attention level (from the lowest average attention at the top to the highest at the bottom). Each column corresponds to four waves. Color shades (from light yellow to dark gray) indicate attention scores: lower attention is yellow and higher attention is dark gray. This heat map shows that some individuals have steady high attention, while others are constantly in a state of low attention or fluctuation.

### 3.2 Average effects: what the conventional approach misses

Table 1 reports the linear fixed effects model that estimates the mean intra-individual association between structured instruction and attention. Model 1 corrected only for the time dummy variable, resulting in a coefficient of 0.285 (*p* < 0.001). When model 2 was added, the coefficient decreased to 0.219 (*p* < 0.001) according to the covariates (age, weekly training time, fatigue). Model 3 adds a quadratic term, which is negative and significant (−0.0032, *p* = 0.014), with a turning point around 49.2 on the 0–100 scale, indicating diminishing marginal returns at higher intensity levels.

If one stops here, the conclusion seems reassuring: structured instruction is, on average, moderately positively associated with attention, although the gains taper off at very high levels of structure. However, the average association masks a key problem. The within-group *R*^2^ for Model 2 is only 0.184, meaning that 82% of the within-individual variance in attention remains unexplained. This lower explanatory power, combined with the broad attention distribution observed in Table 3, suggests that the average association may not generalize well for many individuals. Therefore, we turned to quantile regression to reveal exactly where this association occurred.

### 3.3 Quantile-specific effects

Figure 3 (Sankey diagram) shows a non-parametric preview of heterogeneity by visualizing the flow of attention categories (low, medium, and high) from the pretest (wave 1) to the posttest (wave 4). Among participants with a low starting category, only 18% moved to medium and only 6% reached high. In contrast, 72% of those who started in the high category remained at the high level, and 21% moved to the middle (still above the low). Critically, almost no one went from high to low. This asymmetric mobility suggests that structured instruction, which varies naturally from club to club and wave to wave, helps maintain or slightly improve already high attention, but does little to improve low attention. The Sankey diagram thus sets the stage for a formal quantile analysis.

**Figure 3:**
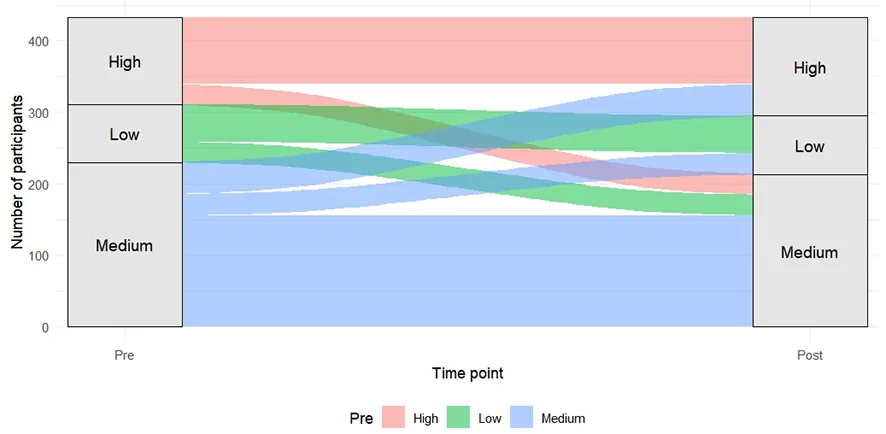
Sankey diagram showing the flow of attention categories from the pretest (wave 1) to the posttest (wave 4). Attention was divided into three categories: low (< 60), medium (60–79), and high (≥ 80). The width of each stream is proportional to the number of participants. The figure shows that the majority of participants with high baseline attention remained at high levels, while participants with low baseline attention had minimal movement to higher categories, indicating limited upward mobility.

Table 2 shows the fixed effects quantile regression coefficients at the τ = 0.10, 0.25, 0.50, 0.75, and 0.90 quantiles. The most striking findings concern structured instruction. At τ = 0.10 (the student with the lowest attention), the coefficient is small and statistically insignificant (0.082, SE = 0.067, *p* = 0.221). At τ = 0.25, the coefficient becomes significant (0.147, *p* = 0.011). At the median (τ = 0.50), the coefficient is 0.239 (*p* < 0.001). The coefficient rises to 0.375 (*p* < 0.001) at τ = 0.75 and reaches 0.506 (*p* < 0.001) at τ = 0.90. The Wald test confirmed a statistically significant difference between τ = 0.10 and τ = 0.90 (χ^2^(1) = 22.47, *p* < 0.001). After a statistical adjustment called the “Benjamini-Hochberg FDR,” the conclusions that originally showed significance did not change, and the results were still reliable.

In particular, we use the “quantile regression” method to look at the strength of the correlation between teaching structure and attention at different levels of attention, rather than dividing athletes into two fixed groups of “high attention” and “low attention.” Therefore, our results reflect the changing laws of each person in different States, rather than stable differences between people. Specifically, when athletes are in the state of low attention, the positive correlation between the perceived teaching structure and attention will be weaker, and when they are in the state of high attention, the positive correlation will be stronger.

Why doesn't perceived structured instruction show a positive association at the lower end of attention? One possibility is that those low-attention States are precisely the moments when athletes tend to zone out or become less engaged while being coached. Even if the training is well organized, they may not be able to absorb it effectively. Another possibility is that athletes in this state find the training structure too rigid and boring, and are more reluctant to participate, while those with high attention find the structure helpful. The following analysis will try to do some exploratory checks from these perspectives.

To facilitate interpretation of practical implications, we calculated the predicted change in attention scores associated with a 10-point increase in perceived structured instruction intensity. At τ = 0.10, the predicted change was 0.82 points (95% CI [-0.52, 2.16]); at τ = 0.50, the predicted change was 2.39 points (95% CI [1.36, 3.42]); at τ = 0.90, the predicted change was 5.06 points (95% CI [3.65, 6.47]). These estimates suggest that the same increase in instructional intensity is associated with a significant increase in the upper quantile of attentional function.

### 3.4 Robustness checks

To test whether our results are reliable, we changed a statistical method (that is, moment-based quantile regression (MMQR) proposed by Machado and Santos Silva, 2019) and re-estimated it. The results showed that the positive association between instructional structure and attention increased as the level of attention increased from low to high, which was consistent with the main fixed-effects quantile regression pattern (see Supplementary Table 4). In addition, considering that the athletes in the data belong to different clubs, we also added the fixed effect of “club X test rounds” and re-analyzed the linear fixed effect model. The results showed that the coefficient of “perceived structured teaching” remained stable (β = 0.208, *p* < 0.001). However, because we only have 8 clubs and the sample size is small, if we make clustering standard errors at the club level, the statistical results are prone to instability, so we finally use the clustering at the individual level as the main basis for inference.

## 4 Discussion

Using fixed-effects quantile regression, this longitudinal study examined whether the association between structured instruction and attention among young badminton players was homogeneous across the distribution of attention. We found a significant pattern of differentiation: the perceived positive intrapersonal association of structured teaching was almost negligible in the observation of the lowest conditional quantile attention (τ = 0.10), but large and significant in the observation of the highest conditional quantile (τ = 0.90). It is important to note that quantile regression estimates associations at different points of the conditional distribution, rather than identifying fixed subgroups of low and high attention individuals. Thus, our results reflect distributional heterogeneity of within-person associations rather than stable person-to-person differences. In the following sections, we integrate the findings with relevant theoretical perspectives, discuss their theoretical and practical implications, acknowledge the study limitations, and outline future research directions.

### 4.1 Core findings, underlying mechanisms, and comparisons with previous literature

#### 4.1.1 Differentiated linkages

The monotonically increasing quantile coefficients from τ = 0.10 to τ = 0.90 (Table 2, Figure 4) indicate that the perceived positive within-person association of structured instruction and attention is stronger at the upper conditional quantile of attentional function. This pattern can be explained by cognitive load theory (Mguidich et al., 2024). When athletes are functioning at higher conditional quantiles of attention, they may have more available cognitive resources to process step-by-step instructions and use structured routines as scaffolds for skill execution. By contrast, when athletes are functioning at lower conditional quantiles, they may be less able to encode or use structured instructions effectively because of distraction, reduced engagement, or limited attentional availability. Similar “expertise reversal effects” in instructional design have been documented: instructional strategies that are effective for novices may be ineffective or even counterproductive for more knowledgeable learners (Mguidich et al., 2024). Our study extends this concept from intellectual expertise to attentional competence. Although previous studies of physical education pedagogy have generally reported average benefits of structured instruction (Coterón et al., 2024; Song et al., 2026), but these studies did not examine distributional effects. Our quantile results suggest that their mean estimates are likely to overestimate the benefits for observations at lower conditional quantiles and underestimate the benefits for observations at upper conditional quantiles. This difference in approach (based on the mean vs. based on the quantile) explains why our conclusions differ from the claim of “uniform validity.”

**Figure 4:**
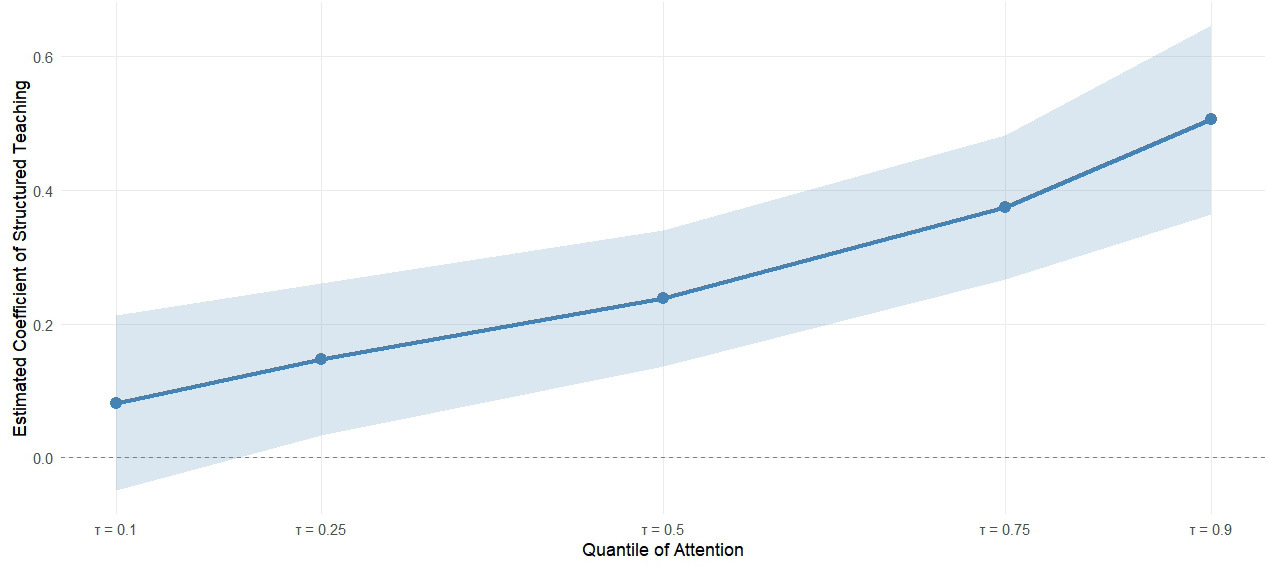
Fixed effects quantile regression coefficients of structured instruction intensity on the distribution of attention. The x-axis shows the conditional quantile of attention (τ), which ranges from 0.10 to 0.90; the y-axis shows the estimated coefficients of structured instruction. Points represent point estimates; shaded bands represent 95% confidence intervals (bootstrap method). The strongly rising slope indicates heterogeneity in the association: the coefficient for structured instruction on the 10th quantile (low attention) is negligible, but the coefficient on the 90th quantile (high attention) is large and significant.

In order to explore the characteristics of athletes at lower levels of attention, we did a tentative restricted cubic spline analysis (for the 25th percentile). Graphically, the results showed a non-linear trend, but it was not clearly confirmed statistically (see Supplementary Figure 1). The formal “change point” test also did not reach statistical significance (*p* = 0.093). Therefore, we do not have enough statistical evidence for the existence of a real “critical value.” This graphical model can only be used as a reference to generate research ideas, and readers should not use it as a reliable basis for segmentation. Confirmatory studies with larger samples and pre-registered analysis protocols are needed before drawing any conclusions about the cutoff value. At present, this trend can only be said to be a preliminary hint that athletes with low attention may need enough “dose” of structured training to overcome attention inertia, but this needs to be tested by specially designed experiments.

One possible mechanism is the “optimal challenge” hypothesis (Tindall-Ford et al., 2019): when instructional structure is low, athletes in low-attention states may experience the training environment as insufficiently engaging or difficult to organize cognitively; When the intensity exceeds a hypothetical critical point, regular training is predictable and structured enough without being boring, thus reducing the cognitive burden. This explanation is consistent with research on attention training in developmental psychology, which shows that children with attention problems tend to benefit from high-intensity, highly structured interventions (Kalyuga, 2014). However, previous studies in youth racket sports have not quantified a specific threshold. Therefore, our exploratory observation only suggests a direction worth studying, not a definite conclusion.

Regarding the matching of “dose” to individuals, we also made an exploratory three-dimensional surface plot (Supplementary Figure 2). This figure shows that the association between perceived structured teaching and the 75th percentile of attention is influenced by the interaction between the intensity of teaching and the fluctuation of athletes‘own attention (that is, the standard deviation of individual attention). Since this surface is based on simulation and smoothing techniques, it is again only exploratory and heuristic. Specifically, for those athletes whose attention fluctuates slightly (that is, their attention is relatively stable), the structured teaching of moderate intensity can bring positive correlation; while for those athletes whose attention fluctuates greatly (that is, their state is very unstable and their fluctuation is obvious), the structure of low intensity has negative correlation, but the structure of high intensity shows strong positive correlation. This pattern can be understood in terms of the theory of attentional resource allocation (Izaki and Ogawa, 2023). Unlike previous studies that treated structured teaching as a “one-size-fits-all” intervention, our results suggest that the intensity of teaching should be adjusted individually according to the stability of students'attention, which of course needs to be further verified by experimental design. This view is consistent with the core principles of adaptive teaching (Yang, 2025) and is supported by recent empirical studies, such as personalized and dose-matched physical activity programs for cognitive functions (including attention), which are more effective than non-adaptive programs (Tao et al., 2025). These observations collectively point toward a preliminary theoretical model that warrants formal articulation.

Taken together, these findings can be understood in terms of the matching of attentional resources. When learners have sufficient attention resources, structured teaching can help learners better control their actions by means of clear routine processes, clear cues and systematic feedback. In this case, the teaching structure can reduce the ambiguity in the task and avoid unnecessary cognitive burden. So that athletes can pay more attention to the key information related to the movement (Kalyuga, 2014; Sweller et al., 2019). This may explain why the association is most pronounced at the higher quantiles of the conditional distribution. On the contrary, at the lower end of the conditional distribution, athletes may be in a state of less attention resources, more easily distracted or less engaged, in which even if the teaching structure is increased, it may be difficult to be effectively absorbed or translated into better attention performance. Thus, the same teaching structure can be a useful scaffold in some States of attention, but may not be sufficient in others, or may instead become an overly restrictive guide. This interpretation is also consistent with some views in the field of motor learning, which hold that too much or mismatched guidance can limit learners' autonomy, prevent them from adopting external focus of attention, reduce opportunities for exploration, and interfere with the flexible coordination between perception and action (Wulf and Lewthwaite, 2016; Renshaw et al., 2019). Therefore, the findings of this study are not intended to overturn existing theories of physical education, but to refine them, pointing out that the value of structured teaching depends not only on how much structure is provided, but also on the current attention state of the learner in the conditional distribution.

### 4.2 Theoretical and practical implications

The theoretical significance of this finding is that it modifies the common view that structured teaching is always beneficial in youth sports training. The results do not support a universal association that applies to all attentional States, but rather suggest that the relationship between instruction and attention varies with different quantiles of the conditional distribution. Therefore, we regard these findings as a preliminary explanation for the matching of attention resources, rather than a proven mechanism. According to this explanation, when athletes have enough attention resources to use routine processes, cues and feedback as support in training, the perceived teaching structure may be of greatest benefit, which is consistent with cognitive load theory and relevant research on teaching design (Kalyuga, 2014; Tindall-Ford et al., 2019). When attention resources are insufficient or unstable, the same teaching structure may be less effective unless it is accompanied by strategies to stabilize participation, reduce distraction or increase the importance of tasks. This explanation expands the perspective of the current field of physical education teaching, focusing not only on whether structured teaching is beneficial in the average sense, but also on under what circumstances, for which people, and in what state of attention, it has the strongest relationship with the function of attention. This also coincides with the view of adaptive teaching, which emphasizes that teaching support should be matched with the needs of learners rather than adopting a unified form of teaching (Yang, 2025). In the future, experimental and procedural studies are needed to directly test this mechanism.

#### 4.2.1 Practical significance

Given the observational nature of this study, these implications are preliminary and should be viewed as hypothesis-generating rather than prescriptive. For coaches and educators of youth badminton, our results suggest not to adopt a uniform and unchanged structured teaching method (Yan et al., 2025). For athletes who already exhibit relatively stable attentional functioning, increasing construct strength is positively associated with attention and may further enhance performance. For students with low attention or high variability, increasing construct intensity alone may not be effective or even be associated with negative outcomes unless the intensity reaches a high level (although this threshold has not been statistically confirmed). For the latter group of students, a more practical strategy is to first develop attentional capacity through attention-training exercises (e.g., mindfulness, response training with change cues; Chen et al., 2024) before introducing a highly structured technique course. Another way is that instead of imposing a rigid framework from the beginning, the coach can adopt an adaptive strategy: start with a medium structure and gradually increase the strength of the structure as the student's attention stabilizes. Our three-dimensional surface suggests that for students with high baseline variability, a low construct may be detrimental; therefore, coaches who instruct adolescents who are easily distracted or hyperactive, either employ a high construct or use other alternative strategies (e.g., gamification, frequent novelty) rather than a partial construct.

### 4.3 Limitations and future directions

There are several limitations in this study. The first and most important point is that this observational longitudinal study design cannot prove causality. Although we used the individual fixed effect model to control for all the confounders that do not change over time, the estimation results may still be affected by some unmeasured factors that change over time, such as the fluctuation of athletes‘motivation, the change of parents' support, or the enthusiasm of coaches. Therefore, all the associations we report can only be understood as correlations between individuals at different time points, not causal relationships. In order to make causal judgments, we need to carry out randomized controlled experiments to manipulate teaching intensity. Second, maturation may act as a source of time-varying confounders. Participants were between the ages of 12 and 16, when attentional control and self-regulation were still developing. Although we used age as a time-varying covariate in our analysis and used indicators at different time points to consider common temporal trends, However, these adjustments do not fully distinguish the perceived structured teaching from the increased attention brought about by the normal maturation of adolescence (Caemmerer et al., 2024). Future research should include more detailed developmental indicators, such as adolescent development, sleep status, and broader self-regulation development, to better distinguish teaching-related effects from maturation-related trajectories of change. Thirdly, the two scales we used (YBARS and STIQ), although validated in an independent sample (see Section 2.5), are, after all, newly developed self-rating instruments. Relying exclusively on self-assessment data introduces the problem of “common method variation,” that is, because all data come from the same person, the observed association may be exaggerated by the same raters (refer to Podsakoff et al.). In the future, objective methods such as eye tracking, reaction time fluctuation, continuous performance test should be added to the research, or coaches should be invited to score, so as to verify from multiple perspectives. In addition, independent cross-validation is needed in other cultural contexts. Fourth, the previous exploratory graphs and three-dimensional surface graphs are only preliminary exploration results. The formal change-point test did not reach statistical significance (*p* = 0.093), and the confidence interval for the breakpoint was wide. Therefore, these figures can only be used as clues to put forward hypotheses, not as definite conclusions. Confirmatory studies with larger samples and pre-registered analysis protocols are needed before recommending specific segmentation points to actual teaching staff. Fifth, we only used a single item to measure fatigue, which is not as reliable as the multi-item scale, so all the results related to fatigue can only be regarded as preliminary. Sixth, the sample of this study is from a city in China (Hengyang), which may limit the applicability of the results to other cultural or educational backgrounds. Seventhly, we only followed for 12 weeks, which may not be enough to capture the longer term dynamics. For example, it is uncertain whether athletes who are in the low attention range will ultimately benefit from more structured training. Eighth, we take “perceived structured instruction” as an overall indicator, but future research can break it down into more specific components, such as clarity, routine processes, feedback methods, etc., in order to find out which specific elements work at different levels of attention. Finally, regarding the nested structure of athletes belonging to different clubs, we have made partial control by adding the fixed effect of “club X test round,” but because there are only eight clubs, the results of clustering robust standard error at the club level are unstable, and the interpretation needs to be cautious. Despite these shortcomings, this study provides the first new evidence of individual differences in the association between teaching and attention, and lays a foundation for future adaptive, dose-matched teaching methods.

## 5 Conclusion

This study shows that there is significant heterogeneity in the association between structured instruction and attention in youth badminton. Linear fixed-effects models that estimate average associations may lead to the potentially misleading conclusion that structured instruction is similarly associated with slight to moderate benefits for all students. In fact, we found that the positive association between teaching structure and attention was significantly stronger when athletes were in a state of high attention level, and significantly weaker when they were in a state of low attention level. Although there is no statistical confirmation, from the exploratory graph, athletes in a state of low attention may need to undergo structured training of sufficient intensity to see a positive association. This differentiated model suggests that if we adopt a unified teaching method for all athletes, we may risk reinforcing attentional differences between them. Coaches may consider assessing each athlete's attention profile first, and then matching the appropriate teaching “dose” accordingly. However, due to the observational design of this study, these suggestions are only preliminary and need to be further verified in the experiment. Future research should examine adaptive interventions and cross-validate these findings in different cultural contexts. Ultimately, promoting the development of physical education pedagogy needs to go beyond the average effect to accept and understand heterogeneity.

## Data Availability

The raw data supporting the conclusions of this article will be made available by the authors, without undue reservation.

## References

[B1] Allison P. (2009). Fixed Effects Regression Models. Thousand Oaks, CA, USA: SAGE Publications, Inc. doi: 10.4135/9781412993869

[B2] Boateng G. O. Neilands T. B. Frongillo E. A. Melgar-Quiñonez H. R. Young S. L. (2018). Best practices for developing and validating scales for health, social, and behavioral research: a primer. Front. Public Health 6:149. doi: 10.3389/fpubh.2018.00149 29942800 PMC6004510

[B3] Brown D. J. Fletcher D. (2017). Effects of psychological and psychosocial interventions on sport performance: a meta-analysis. Sports Med. 47, 77–99. doi: 10.1007/s40279-016-0552-7 27241124

[B4] Brownstein C. G. Rimaud D. Singh B. Fruleux-Santos L. A. Sorg M. Micklewright D. . (2021). French translation and validation of the rating-of-fatigue scale. Sports Med. Open 7:25. doi: 10.1186/s40798-021-00316-8 33829336 PMC8026791

[B5] Caemmerer J. M. Hennessy B. Niileksela C. R. (2024). Third variables in longitudinal research: application of longitudinal mediation and moderation in school psychology. J. School Psychol. 103:101283. doi: 10.1016/j.jsp.2024.101283 38432732

[B6] Cameron A. C. Miller D. L. (2015). A practitioner's guide to cluster-robust inference. J. Hum. Resour. 50, 317–372. doi: 10.3368/jhr.50.2.317

[B7] Canay I. A. (2011). A simple approach to quantile regression for panel data. Econom. J. 14, 368–386. doi: 10.1111/j.1368-423X.2011.00349.x

[B8] Chen L. (2024). Two-step estimation of quantile panel data models with interactive fixed effects. Econom. Theory 40, 419–446. doi: 10.1017/S0266466622000366

[B9] Chen L. Du B. Li K. Li K. Hou T. Jia F. . (2024). The effect of tDCS on inhibitory control and its transfer effect on sustained attention in children with autism spectrum disorder: an fNIRS study. Brain Stimulation 17, 594–606. doi: 10.1016/j.brs.2024.04.019 38697468

[B10] Cohen J. (2013). Statistical Power Analysis for the Behavioral Sciences. New York, NY: Routledge. doi: 10.4324/9780203771587

[B11] Coterón J. Fernández-Caballero J. Martín-Hoz L. Franco E. (2024). The interplay of structuring and controlling teaching styles in physical education and its impact on students motivation and engagement. Behav. Sci. 14:836. doi: 10.3390/bs14090836 PMC11428318 39336051

[B12] Derryberry D. Reed M. A. (2002). Anxiety-related attentional biases and their regulation by attentional control. J. Abnormal Psychol. 111, 225–236. doi: 10.1037/0021-843X.111.2.225 12003445

[B13] Diamond A. Lee K. (2011). Interventions shown to aid executive function development in children 4 to 12 years old. Science 333, 959–964. doi: 10.1126/science.1204529 PMC3159917 21852486

[B14] Dunn J. G. H. Bouffard M. Rogers W. T. (1999). Assessing item content-relevance in sport psychology scale-construction research: issues and recommendations. Meas. Phys. Educ. Exercise Sci. 3, 15–36. doi: 10.1207/s15327841mpee0301_2

[B15] Fung S.-F. Kong C. Y. W. Huang Q. (2019). Evaluating the dimensionality and psychometric properties of the brief self-control scale amongst Chinese university students. Front. Psychol. 10:2903. doi: 10.3389/fpsyg.2019.02903 31969852 PMC6960225

[B16] Gómez-López M. Chicau Borrego C. Marques da Silva C. Granero-Gallegos A. González-Hernández J. (2020). Effects of motivational climate on fear of failure and anxiety in teen handball players. Int. J. Environ. Res. Public Health 17:592. doi: 10.3390/ijerph17020592 31963331 PMC7013665

[B17] Howie E. K. Straker L. M. (2016). Rates of attrition, non-compliance and missingness in randomized controlled trials of child physical activity interventions using accelerometers: a brief methodological review. J. Sci. Med. Sport 19, 830–836. doi: 10.1016/j.jsams.2015.12.520 26874648

[B18] Hu L. Bentler P. M. (1999). Cutoff criteria for fit indexes in covariance structure analysis: conventional criteria versus new alternatives. Struct. Equation Model. Multidiscip. J. 6, 1–55. doi: 10.1080/10705519909540118

[B19] Izaki T. Ogawa K. (2023). Dispersing attentional resources reduces negative emotions. Neuroreport 34, 599–605. doi: 10.1097/WNR.0000000000001929 37395221

[B20] Jin P. Zhao Z.-Q. Zhu X.-F. (2023). The relationship between sport types, sex and visual attention as assessed in a multiple object tracking task. Front. Psychol. 14:1099254. doi: 10.3389/fpsyg.2023.1099254 36910748 PMC9996032

[B21] Junna L. Moustgaard H. Martikainen P. (2022). Current unemployment, unemployment history, and mental health: a fixed-effects model approach. Am. J. Epidemiol. 191, 1459–1469. doi: 10.1093/aje/kwac077 PMC9347014 35441659

[B22] Kalyuga S. (2014). “The expertise reversal principle in multimedia learning,” in The Cambridge Handbook of Multimedia Learning. 2nd edn, ed. R. E. Mayer (Cambridge University Press), 576–597. doi: 10.1017/CBO9781139547369.028

[B23] Kenworthy L. Verbalis A. Bascom J. daVanport S. Strang J. F. Pugliese C. . (2022). Adding the missing voice: how self-report of autistic youth self-report on an executive functioning rating scale compares to parent report and that of youth with attention deficit hyperactivity disorder or neurotypical development. Autism Int. J. Res. Practice 26, 422–433. doi: 10.1177/13623613211029117 34238038 PMC8742839

[B24] Koo T. K. Li M. Y. (2016). A guideline of selecting and reporting intraclass correlation coefficients for reliability research. J. Chiropractic Med. 15, 155–163. doi: 10.1016/j.jcm.2016.02.012 PMC4913118 27330520

[B25] Lee K. J. Carlin J. B. Simpson J. A. Moreno-Betancur M. (2023). Assumptions and analysis planning in studies with missing data in multiple variables: moving beyond the MCAR/MAR/MNAR classification. Int. J. Epidemiol. 52, 1268–1275. doi: 10.1093/ije/dyad008 PMC10396404 36779333

[B26] Liang W. Wang D. D. Shang B. R. Zhang C.-Q. Duan Y. P. Si G. Y. (2022). Further examination of the psychometric properties of the brief self-control scale: evidence from Chinese athletes and students. Int. J. Sport Exercise Psychol. 20, 16–35. doi: 10.1080/1612197X.2020.1827000

[B27] Luong R. Flake J. K. (2023). Measurement invariance testing using confirmatory factor analysis and alignment optimization: a tutorial for transparent analysis planning and reporting. Psychol. Methods 28, 905–924. doi: 10.1037/met0000441 35588078

[B28] Machado J. A. F. Santos Silva J. M. C. (2019). Quantiles via moments. J. Econ. 213, 145–173. doi: 10.1016/j.jeconom.2019.04.009

[B29] McNeish D. (2026). A practical guide to selecting and blending approaches for clustered data: clustered errors, multilevel models, and fixed-effect models. Psychol. Methods 31, 225–251. doi: 10.1037/met0000620 37956085

[B30] Mguidich H. Zoudji B. Khacharem A. (2024). An expertise reversal effect of imagination in learning from basketball tactics. Psychol. Res. 88, 1691–1701. doi: 10.1007/s00426-024-01954-9 38597970

[B31] Micklewright D. St Clair Gibson A. Gladwell V. Al Salman A. (2017). Development and validity of the rating-of-fatigue scale. Sports Med. 47, 2375–2393. doi: 10.1007/s40279-017-0711-5 28283993 PMC5633636

[B32] Moran A. P. (2016). The Psychology of Concentration in Sport Performers: A Cognitive Analysis, 1st edn. Psychology Press. doi: 10.4324/9781315784946

[B33] Poon K. Ho M. S. H. Wang L. C. Lee H. M. Lau W. K. W. Chan W. W. L. (2024). Improving cognitive function in Chinese children with ADHD and/or RD through computerized working memory training. BMC Psychol. 12:574. doi: 10.1186/s40359-024-02065-1 39425120 PMC11490009

[B34] Putnick D. L. Bornstein M. H. (2016). Measurement invariance conventions and reporting: the state of the art and future directions for psychological research. Dev. Rev. 41, 71–90. doi: 10.1016/j.dr.2016.06.004 PMC5145197 27942093

[B35] Pyke W. Lunau J. Javadi A.-H. (2025). Does difficulty moderate learning? A comparative analysis of the desirable difficulties framework and cognitive load theory. Q. J. Exp. Psychol. 78, 2181–2195. doi: 10.1177/17470218241308143 39641213 PMC12432286

[B36] R Core Team (2025). R: A language and environment for statistical computing (Version 4.4.2). Vienna: R Foundation for Statistical Computing. Available online at: https://www.R-project.org/

[B37] Renshaw I. Davids K. Newcombe D. Roberts W. M. (2019). The Constraints-Led Approach: Principles for Sports Coaching and Practice Design. London: Routledge.

[B38] Sader J. Bajwa N. M. Lüchinger R. Fassier T. Audétat M. C. Nendaz M. . (2026). Script or style? Analysis of the relationship between teaching scripts and supervision style, *PLoS One* 21:e0338902. doi: 10.1371/journal.pone.0338902 41505471 PMC12782432

[B39] Saw A. E. Main L. C. Gastin P. B. (2016). Monitoring the athlete training response: subjective self-reported measures trump commonly used objective measures: a systematic review. Br. J. Sports Med. 50, 281–291. doi: 10.1136/bjsports-2015-094758 26423706 PMC4789708

[B40] Silva A. F. Ramirez-Campillo R. Sarmento H. Afonso J. Clemente F. M. (2021). Effects of training programs on decision-making in youth team sports players: a systematic review and meta-analysis. Front. Psychol. 12:663867. doi: 10.3389/fpsyg.2021.663867 34135822 PMC8200496

[B41] Song X. Wang D. Wang D. Chen C. Wang Z. (2026). The effect of taekwondo structured training on attention in 7–8-year-old children: a randomized controlled trial. BMC Sports Sci. Med. Rehabil. 18:130. doi: 10.1186/s13102-026-01570-7 PMC12990622 41668137

[B42] Sweller J. Van Merriënboer J. J. G. Paas F. (2019). Cognitive architecture and instructional design: 20 years later. Educ. Psychol. Rev. 31, 261–292. doi: 10.1007/s10648-019-09465-5

[B43] Tangney J. P. Baumeister R. F. Boone A. L. (2004). High self-control predicts good adjustment, less pathology, better grades, and interpersonal success. J. Personality 72, 271–324. doi: 10.1111/j.0022-3506.2004.00263.x 15016066

[B44] Tao R. Yang Y. Wilson M. Chang J. R. Liu C. Sit C. H. P. (2025). Comparative effectiveness of physical activity interventions on cognitive functions in children and adolescents with Neurodevelopmental Disorders: a systematic review and network meta-analysis of randomized controlled trials. Int. J. Behav. Nutr. Phys. Activity 22:6. doi: 10.1186/s12966-024-01702-7 39806448 PMC11731537

[B45] Tindall-Ford S. Agostinho S. Sweller J. (2019). Advances in Cognitive Load Theory: Rethinking Teaching. London New York: Routledge (Local/Global Issues in Education Series). doi: 10.4324/9780429283895

[B46] van Andel S. Pieper R. Werner I. Wachholz F. Mohr M. Federolf P. (2022). Implications of optimal feedback control theory for sport coaching and motor learning: a systematic review. Motor Control 26, 144–167. doi: 10.1123/mc.2021-0041 34920414

[B47] Wang C. Li R. Zheng W. (2025). Analogy learning to maintain motor performance stability and its cognitive mechanisms: a comprehensive review. Perceptual Motor Skills 132, 623–645. doi: 10.1177/00315125251330660 40129041

[B48] Wulf G. Lewthwaite R. (2016). Optimizing performance through intrinsic motivation and attention for learning: The OPTIMAL theory of motor learning. Psychon. Bull. Rev. 23, 1382–1414. doi: 10.3758/s13423-015-0999-9 26833314

[B49] Yan J. Wu C. Tan X. Dai M. (2025). The influence of AI-driven personalized foreign language learning on college students mental health: a dynamic interaction among pleasure, anxiety, and self-efficacy. Front. Public Health 13:1642608. doi: 10.3389/fpubh.2025.1642608 41000311 PMC12457127

[B50] Yang C. (2025). Adapting teaching methods to accommodate diverse learning styles in education. J. Higher Educ. Res. 5:535. doi: 10.32629/jher.v5i6.3382

[B51] Zheng H. Xiang Z. Yang W. (2025). How parental involvement in youth sports impacts on school adjustment: a dual mediation pathway via sports interest and extracurricular sports participation. Front. Public Health 13:1621980. doi: 10.3389/fpubh.2025.1621980 40687128 PMC12270873

[B52] Zhu Y. Sun F. Li C. Chow D. H. K. Wang K. (2020). Acute effect of brief mindfulness-based intervention coupled with fluid intake on athletes' cognitive function. J. Sports Sci. Med. 19, 753–760. 33239950 PMC7675633

[B53] Zuber C. Charbonnet B. (2023). Self-report or coach-rating? The predictive validity of different approaches to assess athletes' achievement motivation in team and individual sports. J. Sports Sci. 41, 1645–1653. doi: 10.1080/02640414.2023.2289271 38031810

