# Supplementary material for: Longitudinal associations between structured teaching and attentional functioning in youth badminton: evidence from fixed effects quantile regression
Source: Front Psychol. 2026 Sep 22;17:1908358. doi: 10.3389/fpsyg.2026.1908358 (PMC13638408; doi:10.3389/fpsyg.2026.1908358)
Supplement: Supplementary file 1 [file Table_1.docx]

| Supplementary Table S1. Longitudinal measurement invariance fit indices for YBARS and STIQ across four waves | | | | | | | |
| --- | --- | --- | --- | --- | --- | --- | --- |
| Panel | Model | CFI | RMSEA | SRMR | ΔCFI | ΔRMSEA | Decision |
| A: YBARS | Configural | 0.942 | 0.058 | 0.045 | — | — | Acceptable |
|  | Metric | 0.938 | 0.059 | 0.048 | -0.004 | +0.001 | Supported |
|  | Scalar | 0.926 | 0.063 | 0.055 | -0.012 | +0.004 | Not supported |
|  | Partial Scalar (Item 5 freed) | 0.936 | 0.060 | 0.049 | -0.006 | +0.002 | Supported |
| B: STIQ | Configural | 0.931 | 0.061 | 0.052 | — | — | Acceptable |
|  | Metric | 0.927 | 0.062 | 0.055 | -0.004 | +0.001 | Supported |
|  | Scalar | 0.915 | 0.067 | 0.062 | -0.016 | +0.005 | Not supported |
|  | Partial Scalar (Item 3 & 9 freed) | 0.925 | 0.063 | 0.057 | -0.006 | +0.002 | Supported |
| Note. ΔCFI ≤ -0.01 and ΔRMSEA ≥ 0.015 indicate a significant degradation in fit. The partial scalar invariance models （with freed intercepts） were accepted, supporting the comparability of latent constructs across the four waves. | | | | | | | |
