# Supplementary material for: Longitudinal associations between structured teaching and attentional functioning in youth badminton: evidence from fixed effects quantile regression
Source: Front Psychol. 2026 Sep 22;17:1908358. doi: 10.3389/fpsyg.2026.1908358 (PMC13638408; doi:10.3389/fpsyg.2026.1908358)
Supplement: Supplementary file 2 [file Table_2.docx]

| Supplementary Table S2. Factor loadings, convergent reliability, and internal consistency of the YBARS and STIQ (N = 433) | | | | | | |
| --- | --- | --- | --- | --- | --- | --- |
| Panel | Dimension / Item | Std. CFA Loading | Cronbach's α | McDonald's ω | CR | AVE |
| A: YBARS | Sustained Attention |  | 0.87 | 0.88 | 0.86 | 0.56 |
|  | YBARS_1: Focus on coach's explanation | 0.74 |  |  |  |  |
|  | YBARS_3: Maintain focus during repeated drills | 0.81 |  |  |  |  |
|  | YBARS_5: Concentrate without mind-wandering | 0.68 |  |  |  |  |
|  | YBARS_8: Stay on task despite fatigue | 0.76 |  |  |  |  |
|  | Selective Attention |  | 0.85 | 0.86 | 0.84 | 0.54 |
|  | YBARS_2: Filter out court distractions | 0.72 |  |  |  |  |
|  | YBARS_6: Attend to ball amidst noise | 0.79 |  |  |  |  |
|  | YBARS_9: Ignore irrelevant movements | 0.66 |  |  |  |  |
|  | Attention Switching |  | 0.86 | 0.87 | 0.85 | 0.58 |
|  | YBARS_4: Shift focus between exercises | 0.80 |  |  |  |  |
|  | YBARS_7: Transition attention quickly | 0.75 |  |  |  |  |
|  | YBARS_10: Re-focus after interruptions | 0.69 |  |  |  |  |
| B: STIQ | Instruction Clarity |  | 0.89 | 0.90 | 0.89 | 0.61 |
|  | STIQ_1: Clear step-by-step instructions | 0.82 |  |  |  |  |
|  | STIQ_5: Precise demonstration | 0.78 |  |  |  |  |
|  | STIQ_9: Explicit task goals | 0.71 |  |  |  |  |
|  | Routine Predictability |  | 0.88 | 0.88 | 0.87 | 0.57 |
|  | STIQ_2: Fixed session flow | 0.76 |  |  |  |  |
|  | STIQ_6: Know what comes next | 0.80 |  |  |  |  |
|  | STIQ_10: Consistent training schedule | 0.68 |  |  |  |  |
|  | Systematic Feedback |  | 0.91 | 0.91 | 0.90 | 0.60 |
|  | STIQ_3: Structured feedback after each drill | 0.84 |  |  |  |  |
|  | STIQ_7: Corrective guidance | 0.79 |  |  |  |  |
|  | STIQ_11: Performance review sessions | 0.73 |  |  |  |  |
|  | Progressive Scaffolding |  | 0.88 | 0.89 | 0.87 | 0.55 |
|  | STIQ_4: Skills introduced from basic to complex | 0.77 |  |  |  |  |
|  | STIQ_8: Gradual increase in difficulty | 0.72 |  |  |  |  |
|  | STIQ_12: Logical sequence of tasks | 0.70 |  |  |  |  |
| Note: Confirmatory Factor Analysis (CFA) was conducted on the full sample (N=433) given the theoretically established dimensions. All loadings are standardized and significant at p < 0.001. CR = Composite Reliability; AVE = Average Variance Extracted. | | | | | | |
