# Supplementary material for: Longitudinal associations between structured teaching and attentional functioning in youth badminton: evidence from fixed effects quantile regression
Source: Front Psychol. 2026 Sep 22;17:1908358. doi: 10.3389/fpsyg.2026.1908358 (PMC13638408; doi:10.3389/fpsyg.2026.1908358)
Supplement: Supplementary file 3 [file Table_3.docx]

| **Supplementary Table S3. Discriminant validity (HTMT) and test-retest reliability (ICC) of the YBARS and STIQ** | | | | | | | |
| --- | --- | --- | --- | --- | --- | --- | --- |
| **Panel A. HTMT Ratios for Discriminant Validity (N = 433)** | | | | | | | |
| Construct | 1 | 2 | 3 | 4 | 5 | 6 | 7 |
| 1. YBARS_Sustained | — |  |  |  |  |  |  |
| 2. YBARS_Selective | 0.68 | — |  |  |  |  |  |
| 3. YBARS_Switching | 0.62 | 0.71 | — |  |  |  |  |
| 4. STIQ_Clarity | 0.45 | 0.41 | 0.38 | — |  |  |  |
| 5. STIQ_Predictability | 0.39 | 0.34 | 0.41 | 0.69 | — |  |  |
| 6. STIQ_Feedback | 0.42 | 0.37 | 0.35 | 0.74 | 0.65 | — |  |
| 7. STIQ_Scaffolding | 0.36 | 0.31 | 0.28 | 0.72 | 0.63 | 0.70 | — |
| Note. All HTMT values are below the recommended strict threshold of 0.85 (Henseler et al., 2015), supporting adequate discriminant validity between the YBARS and STIQ dimensions, and between the two overarching constructs. | | | | | | | |
| **Panel B. Test-Retest Reliability (14-day interval, Sub-sample n = 45)** | | | | | | | |
| Scale / Subscale | ICC(2,1) | 95% CI | SEM | Interpretation | | | |
| YBARS Total Score | 0.84 | [0.73, 0.91] | 5.28 | Good temporal stability | | | |
| YBARS_Sustained Attention | 0.83 | [0.71, 0.90] | 5.41 | Acceptable | | | |
| YBARS_Selective Attention | 0.81 | [0.68, 0.89] | 5.75 | Acceptable | | | |
| YBARS_Attention Switching | 0.82 | [0.70, 0.90] | 5.56 | Acceptable | | | |
| STIQ Total Score | 0.87 | [0.78, 0.93] | 5.12 | Good temporal stability | | | |
| STIQ_Instruction Clarity | 0.86 | [0.76, 0.92] | 5.31 | Good | | | |
| STIQ_Routine Predictability | 0.85 | [0.74, 0.92] | 5.50 | Good | | | |
| STIQ_Systematic Feedback | 0.88 | [0.79, 0.93] | 4.87 | Good | | | |
| STIQ_Progressive Scaffolding | 0.84 | [0.72, 0.91] | 5.59 | Good | | | |
| **Note.** ICC = Intraclass Correlation Coefficient (two-way mixed, absolute agreement). SEM = Standard Error of Measurement, calculated as SD × √(1 − ICC), using the pooled baseline SD (YBARS SD = 13.2; STIQ SD = 14.2). The ICC values are significantly superior to the simple Pearson r (all r ≥ 0.82), confirming that these instruments reliably capture stable trait-like variations across time rather than random fluctuations. | | | | | | | |
