# Supplementary material for: Longitudinal associations between structured teaching and attentional functioning in youth badminton: evidence from fixed effects quantile regression
Source: Front Psychol. 2026 Sep 22;17:1908358. doi: 10.3389/fpsyg.2026.1908358 (PMC13638408; doi:10.3389/fpsyg.2026.1908358)
Supplement: Supplementary file 4 [file Table_4.docx]

| Supplementary Table 4. Robustness Check: MMQR Estimates for Perceived Structured Teaching on Attentional Functioning | | | | | |
| --- | --- | --- | --- | --- | --- |
| Quantiles (τ) | Coefficient (β) | Bootstrap S.E. | t-statistic | p-value | [95% Conf. Interval] |
| Q10 | 0.118 | 0.045 | 2.62 | 0.009 | [0.030, 0.206] |
| Q25 | 0.165 | 0.038 | 4.34 | <0.001 | [0.091, 0.239] |
| Q50 | 0.208 | 0.032 | 6.50 | <0.001 | [0.145, 0.271] |
| Q75 | 0.276 | 0.041 | 6.73 | <0.001 | [0.196, 0.356] |
| Q90 | 0.314 | 0.052 | 6.04 | <0.001 | [0.212, 0.416] |
| Observations (N) = 592, Number of individuals (n) = 148, Waves (T) = 4.  Notes: MMQR estimates confirm the monotonically increasing pattern of coefficients across quantiles, consistent with the main findings in Table 3. Numerical differences in the lower tail (τ = 0.10) reflect known sensitivity of quantile estimates in short panels (T = 4) and do not undermine the primary conclusion of distributional heterogeneity. MMQR was estimated on the balanced subsample (n = 148) for computational stability. All models included individual fixed effects, wave fixed effects, and time-varying covariates (age, weekly training hours, fatigue). Standard errors: cluster-bootstrap (500 replications) at the individual level. | | | | | |
